# scTrends: A living review of commercial single-cell and spatial 'omic technologies

**DOI:** 10.1016/j.xgen.2024.100723

**Published:** 2024-12-11

**Authors:** Joachim De Jonghe, James W. Opzoomer, Amaia Vilas-Zornoza, Benedikt S. Nilges, Peter Crane, Marco Vicari, Hower Lee, David Lara-Astiaso, Torsten Gross, Jörg Morf, Kim Schneider, Juliana Cudini, Lorenzo Ramos-Mucci, Dylan Mooijman, Katarína Tiklová, Sergio Marco Salas, Christoffer Mattsson Langseth, Nachiket D. Kashikar, The scTrends Consortium, Eli M. Carrami, Rebecca McIntyre, Casey Benjamin Swerner, Edith M. Hessel, ChantrioInt-Andreas Kapourani, Cristian Regep, Charles E.S. Roberts, Denis Schapiro, Joakim Lundeberg, Mats Nilsson, Alex K. Shalek, Adam P. Cribbs, Jake P. Taylor-King

**Affiliations:** 1 Francis Crick Institute, London, UK; 2 Cell Communication Lab, Department of Oncology, University College London Cancer Institute, London, UK; 3 Relation Therapeutics, London, UK; 4 OMAPiX GmbH, Langenfeld, Rheinland, Germany; 5 Caeruleus Genomics, Oxford, UK; 6 Science for Life Laboratory, Department of Gene Technology, KTH Royal Institute of Technology, Solna, Sweden; 7 spatialist AB, Stockholm, Sweden; 8 Science for Life Laboratory, Department of Biochemistry and Biophysics, Stockholm University, 171 65 Solna, Sweden; 9 Department of Hematology, University of Cambridge, Cambridge, UK; 10 Wellcome-MRC Cambridge Stem Cell Institute, Cambridge, UK; 11 Skyhawk Therapeutics, Basel, Switzerland; 12 Roche, Basel, Switzerland; 13 Single Cell Discoveries, Utrecht, the Netherlands; 14 Institute for Computational Biomedicine, Heidelberg University, Faculty of Medicine, Heidelberg University Hospital, Heidelberg, Germany; 15 Institute of Pathology, Heidelberg University Hospital, Heidelberg, Germany; 16 Translational Spatial Profiling Center (TSPC), Heidelberg, Germany; 17 Institute for Medical Engineering and Science, Department of Chemistry and Koch Institute for Integrative Cancer Research, Massachusetts Institute of Technology, Cambridge, MA, USA; 18 Broad Institute of MIT and Harvard, Cambridge, MA, USA; 19 Ragon Institute of MGH, MIT, and Harvard, Cambridge, MA, USA; 20 Botnar Research Centre, Nuffield Department of Orthopaedics, Rheumatology and Musculoskeletal Sciences, National Institute of Health Research Oxford Biomedical Research Unit (BRU), University of Oxford, Oxford, UK; 21 Oxford Centre for Translational Myeloma Research University of Oxford, Oxford, UK

## Abstract

Understanding the rapidly evolving landscape of single-cell and spatial omic technologies is crucial for advancing biomedical research and drug development. We provide a living review of both mature and emerging commercial platforms, highlighting key methodologies and trends shaping the field. This review spans from foundational single-cell technologies such as microfluidics and plate-based methods to newer approaches like combinatorial indexing; on the spatial side, we consider next-generation sequencing and imaging-based spatial transcriptomics. Finally, we highlight emerging methodologies that may fundamentally expand the scope for data generation within pharmaceutical research, creating opportunities to discover and validate novel drug mechanisms. Overall, this review serves as a critical resource for navigating the commercialization and application of single-cell and spatial omic technologies in pharmaceutical and academic research.

## Graphical abstract

**Figure:**
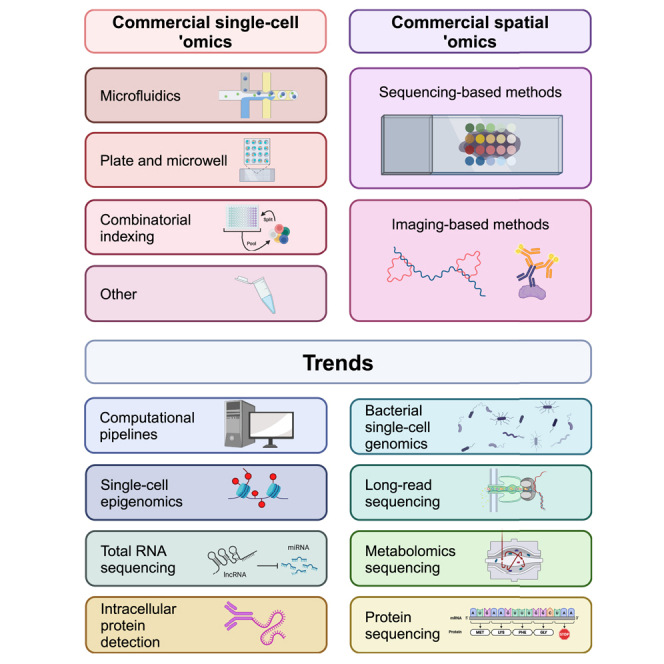

## Teaser

De Jonghe et al. present a comprehensive review of current commercial single-cell and spatial omic technologies, outlining key platforms, methodologies, and trends shaping the field—from microfluidics and plate-based approaches to combinatorial indexing, highlighting their applications in drug discovery. The authors discuss emerging engineering and computational advancements that are enabling more efficient data generation and interpretation. Finally, they explore new tools and techniques that promise to expand the scope of omic analyses, driving innovation and understanding in biomedical research.

## Introduction

Single-cell genomic profiling technologies are becoming the *de facto* standard for studying complex biological and clinical samples, resulting in a multi-billion dollar market.^1^^,^^2^^,^^3^ Currently, a small number of established players, such as 10× Genomics, NanoString Technologies, and Vizgen, have achieved broad acceptance of their technologies through assay reliability, comprehensive technical support, and standardized preprocessing pipelines. However, such technologies are typically associated with high upfront costs for hardware. Early technology development efforts predominantly focused on increasing the number of cells profiled. This approach has been instrumental in discovering rare cancerous and immunological cell types in suspension, often prioritizing cell quantity over sequencing depth. However, there has been a gradual shift in focus toward new applications, such as arrayed chemical and pooled CRISPR screens with single-cell readouts.^4^^,^^5^^,^^6^ These emerging techniques open new paradigms: fewer cells might be targeted, but with greater coverage, to more effectively elucidate dysregulated pathways. In a similar vein, the evolution of spatial profiling technology may also be witnessing a transition. Initially defined by resolution limitations, the current development trajectory is increasingly characterized by the ability to multiplex. A number of groups are now commercializing novel protocols, including hardware-free single-cell systems, and may come to enrich the repertoire of established methodologies. To this end, our objective is to catalog technologies transitioning from academic research to commercial availability, providing a comprehensive resource for the bioscience industry. This endeavor serves a dual purpose: first, to facilitate informed decision-making for potential buyers by offering a detailed overview of commercial technology and, second, to bridge the gap between academic innovation and industry application by highlighting emerging tools.

Remarkably, the field is moving evermore rapidly; so much so that it is challenging for any single practitioner to maintain a working knowledge of the available technologies alongside other research interests. Therefore, we initiated the scTrends consortium^7^ (https://sctrends.org/) to build a “living review”: a community effort that will allow for continual updates as platforms and trends emerge. At this point, scTrends will take the form of a desk review, but in later editions, we plan to benchmark protocols in a standardized manner. Should you wish to contribute, please do not hesitate to get in touch.

In our first release,^7^ we focused on technology application areas, business models, litigation, mergers, and acquisitions before considering public markets. In contrast, this long-form review provides a deep dive into the broad categories of engineering solutions developed for these purposes. To complement this, available at https://sctrends.org/, we present a detailed individual analysis of commercially available single-cell and spatial omic technology platforms. Finally, we comment on breakthrough emerging techniques from academia that we expect to see commercialized in the coming years, ranging from epigenetics, long-read and total RNA sequencing (RNA-seq), bacterial profiling, and metabolomics to intracellular proteomics and protein sequencing.

## Commercial single-cell omic technologies

**Figure 1:**
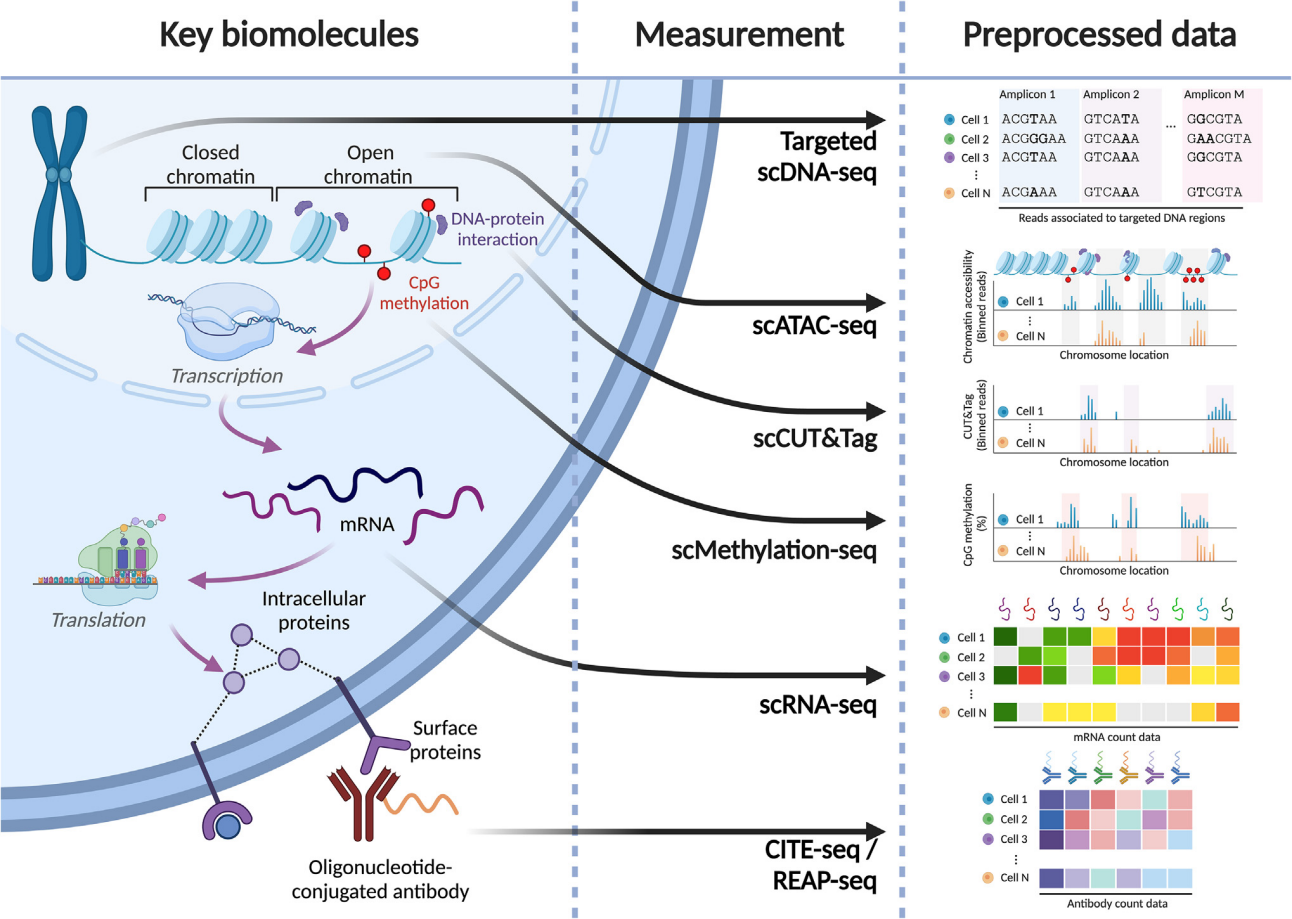
Diagram of key biomolecules measurable at single-cell resolution by commercially available solutions in kit format Adapted from figure contained in Peidli et al.^9^

Since the Human Genome Project, with the aim to characterize a whole human genome, a number of omic technologies have been developed to understand aspects of nucleic acid biology,^8^ e.g., RNA-seq, ATAC-seq, chromatin immunoprecipitation (ChIP)-seq, Hi-C, etc. Only some of these technologies have been adapted to kit-based products at single-cell resolution, including targeted DNA sequencing, ATAC-seq, Cleavage Under Targets and Tagmentation (CUT&Tag), RNA-seq, DNA methylation, and cell surface proteomics, while transcripts and proteins can be measured spatially using a commercial solution. In Figure 1, we plot a diagrammatic overview of the aforementioned commercially available single-cell omic modalities and the subsequent processed output dataset. A number of standard adaptations are widely available, including 5′ mRNA sequencing kits for co-sequencing of V(D)J repertoires from T and/or B cells and capture of guide RNAs from pooled CRISPR screens.

While microfluidic-based methods were the first high-throughput method to receive wide-scale adoption, competing methods emerged; to a broad approximation, we classify these methods into microfluidic-based, plate-based (including via the use of microwells), and combinatorial-indexing-based technologies—in addition to miscellaneous methods that fall outside of this trichotomy. In Tables S1 and S2, we present summary statistics pertaining to the companies below, including other companies that are not detailed due to company stage, focus, or inactivity.

### Microfluidics-based methods

In order to increase throughput from plate-based assays, new methodologies turned to microfluidics to parallelize the number of cells that could be assayed in a single experiment. First, the Fluidigm C1 proposed a large-scale integrated (LSI) system to process up to 96 cells in parallel on a single chip. However, the high cost associated with the method and still relatively low throughput required further technological advancements. Notably, the partitioning capabilities of droplet microfluidics were an attractive prospect for scaling beyond LSIs. inDrop^10^ and Drop-seq,^11^ published in 2015, used water-in-oil emulsions to stochastically co-encapsulate single cells with barcoded oligo(dT) beads to capture and tag the 3′ tail of mRNA molecules from individual cells, linking barcodes to single-cell transcriptomes via cDNA synthesis, marking the advent of high-throughput single-cell genomics.

Since then, many droplet microfluidic setups for different modalities have been published in academic settings, including HyDrop,^12^ spinDrop,^13^ VASA-seq,^14^ MATQ-drop,^15^ droplet ChIP-seq and CUT&Tag implementations,^16^^,^^17^^,^^18^^,^^19^ and snRandom-seq,^20^ among others. However, most assays are run on the 10× Genomics Chromium platform. Large intellectual property portfolios, early adoption, and discounts for large-scale initiatives/users, such as the Human Cell Atlas,^21^ are all factors explaining the dominance of this single player in the field. Although Bio-Rad has proposed its own single-cell implementation (such as the SureCell ATAC-seq library^22^ preparation method), it failed to contend with the 10× Chromium in terms of user adoption. Mission Bio has also emerged as a significant player in the targeted DNA capture field at single-cell resolution. Their Tapestri platform specializes in analyzing the relationship between genomic mutational profiles and expressed phenotypes measured by antibody panels. Unlike other platforms, it does not focus on transcriptomics, thereby setting a distinct offering in DNA-based measurements.

Other instrument companies that deliver single-cell genomics using droplet microfluidics exist, including 1cellbio (now defunct), Scope Fluidics, Dolomite Bio, and Fluigent, but are out of scope for this review, as they do not offer an end-to-end solution for single-cell profiling, are focused on diagnostics, and/or are yet to initiate a commercial offering.

### Plate- and microwell-based methods

In parallel, through reducing well size and simplifying workflows, efforts were made to increase cell throughput and decrease the financial and labor demands associated with processing cells in multiwell plates. To take advantage of the operational simplicity and sample efficiency on well-based approaches relative to droplets,^23^ two avenues were pursued.

On the one hand, akin to the aforementioned LSI approaches, increasingly sophisticated microfluidic technologies were built to facilitate the deterministic manipulation of individual cells toward supplanting a reliance on manual or fluorescence-activated cell-sorting-based methods for single-cell isolation.^24^^,^^25^ These solutions also reduce reagent costs and hands-on processing time. This approach is embodied by the Takara ICELL8 cx Single-Cell System, which supports the processing of 5,184 nanowells in parallel and can be leveraged to implement several different molecular protocols (e.g., SMART-seq-based single-cell RNA-seq [scRNA-seq], single-cell ATAC-seq [scATAC-seq], and single-cell TCR-seq, as well as user-designed protocols).

On the other hand, strategies were developed that relied on probabilistic pairing of barcoded beads with single cells in massively parallel picowell arrays. These efforts were intellectually akin to those pursued in parallel in droplets, with the picowell replacing the droplet as the partition.^26^ Earliest efforts have relied on co-confining cells and beads in unsealed nanowell arrays, as in CytoSeq, which was later refined and commercialized by BD as its Rhapsody system. Note that a similar approach underlies Microwell-Seq.^27^

However, the use of an open well design has the potential to considerably limit capture efficiency and increase cross-contamination between wells. To overcome this barrier, approaches were developed to seal the picowell arrays within microfluidic chips with an oil barrier.^28^ One instantiation of this strategy was commercialized by Celsee via their Genesis System,^29^ which is now offered by Bio-Rad following their acquisition; another implementation, scFTD-seq,^30^ has since been commercialized by Singleron via their SCOPE-chip. As an alternative, semipermeable, membrane-based seals were devised. In some embodiments, this was shown to enable fluid exchange, opening a route to more flexible molecular manipulation via multi-step protocols.^23^ Membrane-based sealing has been used for high-throughput repertoire profiling^31^ and, in a simplified deployment, for scRNA-seq in resource-limited settings around the world^23^^,^^32^ (Seq-Well). The latter technology has since been further simplified and commercialized as the HIVE by Honeycomb Biotechnologies.

### Combinatorial indexing methods

Offered by Parse Biosciences and Scale Biosciences, combinatorial-indexing-based sequencing assays were developed as a strategy to tackle the often prohibitively high upfront hardware investment and library construction costs that could be associated with assaying transcriptomic cellular heterogeneity with a view toward tissue scale profiling.^33^^,^^34^^,^^35^^,^^36^ At a high level, combinatorial indexing methods use fixed and permeabilized cells (or nuclei) as the principal reaction container to iteratively append DNA barcodes to a cell’s RNA-derived reverse-transcribed cDNA through the process of repeatedly pooling the cells and partitioning them randomly across a series of wells containing the oligonucleotide barcodes. Through this repeated process, as the number of wells and barcoding rounds increase, the number of unique barcode combinations available for any single-cell experiment becomes extremely large. When the input cells into the assay are a small fraction of the total possible barcode space (∼5%), the vast majority of cells acquire a unique barcode combination, facilitating the demultiplexing of cells after sequencing. This often results in a lower “multiplet” rate when compared to the more popular microfluidic-based technologies. However, it is crucial to acknowledge the challenges associated with split-pool barcoding, such as the increased complexity in barcode assignment and potential errors in barcode synthesis or sequencing, which can impact the accuracy and efficiency of the method.

As combinatorial indexing has matured, its application has extended beyond scRNA-seq. It is now a versatile tool for both single-cell atlases and functional genomics applications, including chemical transcriptomics-based drug screening^37^ and pooled single-cell CRISPR screening.^38^ These classes of protocols have a built-in capacity to multiplex 96 or more samples based on the number of unique barcodes generated in the initial barcoding round within a 96-well plate setup. Consequently, it facilitates the processing of larger numbers of cells per barcoding run, potentially sequencing more than ∼1 million cells in a single experiment. The capacity is about not merely enabling a greater sequencing scale but also fundamentally reducing preparation costs prior to sequencing. In comparison to other methods, combinatorial indexing offers a more cost-effective approach during the preparation stage, making it a more efficient choice for large-scale genomic studies. Moreover, since these methods leverage fixed sample material, cells or nuclei can be frozen and stored before running the protocol, allowing for asynchronous sample collection before running and reduction in potential confounding batch effects. Often, these methods eliminate the requirement for specialized instrumentation like microfluidics or micropatterned plates, thus enhancing the accessibility of the protocol compared to many others.

Understanding the specific contexts in which these methods are most effective is essential. For instance, there is a significant amount of cell loss during the barcoding stage due to the transfer of cells across multiple 96-well plates, making it unsuitable for very small samples with limited cell availability. However, this can be overcome through sample multiplexing across many individual samples where samples are limited, and in preliminary exploratory cohort studies, combinatorial indexing methods can be particularly advantageous. It is also important to acknowledge the challenges in practical implementation. The protocol involves multiple, key, manual steps in which precision is crucial and errors can occur. These complexities can make the procedure demanding and may require a level of expertise and practice. While multiplexing 96 samples is a step change in multiplexing scales compared to droplet microfluidic platforms, it comes with practical challenges at the bench. As development continues on these methods, they will benefit greatly from advances in accessible laboratory automation, which could enable further increases in the scale of single-cell data-generation efforts going forward.

### Miscellaneous methods

To extend the reach of single -ell technologies to users without access to well-equipped genomic core facilities, there is a need to simplify single-cell library preparation of laborious workflows (such as with combinatorial indexing) and to reduce upfront instrument costs (such as with microfluidic approaches) while maintaining a higher throughout of cells than plate-based solutions. Thus, alternative instrument-free methods have been developed by a number of academic groups and companies. Fluent BioSciences’ PIPseq platform encapsulates single cells and barcoded particles into water-in-oil droplets through a simple vortexing step. Their particle-templated emulsification generates uniformly sized droplets similar to microfluidic devices, and droplet generation is compatible with a range of formats, including microwell plates for high sample numbers or larger conical tubes to sample up to millions of cells. Scipio Bioscience developed their Asteria platform, which differs from other methods in that the impenetrable separation between cells in droplets and plate wells is replaced by a local confinement of individual cells together with barcoded beads in a reversible hydrogel. Cell lysis is performed after the hydrogel transitions from a liquid to a gel state. While liberated RNA molecules might diffuse at slow rates through gels, hybridization of RNAs to DNA barcodes is much faster and enables clean single-cell transcriptome capture. Scipio Bioscience’s approach has been adopted by CS Genetics, which recently made public the upcoming launch of their platform applying reversible gelation to capture single cells on barcoded particles.

## Commercial spatial omic technologies

If scRNA-seq allows disentangling cellular transcriptomic heterogeneity among individual cells within a sample, it lacks the capability to elucidate the spatial context of these transcripts or cells within the tissue. The positioning of cells and their interactions within a specific tissue environment can influence their gene expression patterns and, consequently, their roles in health and disease.

Traditional tissue dissociation methods, used to prepare samples for scRNA-seq, often lead to the loss of spatial information and can perturb the native state of cells. Some cell types are difficult to extract efficiently, and dissociation can alter their gene expression profiles, resulting in an incomplete or biased representation of tissue heterogeneity.^39^ Some of these issues can be somewhat negated using nuclei, but limitations around the incomplete capture of the cellular transcriptomes underscore the need for techniques that preserve the spatial context of cells and their native interactions.^40^^,^^41^ To address this gap, a range of technologies, collectively termed “spatial omics,” have been developed. As a characterized framework proposed by Williams et al.*,*^42^ these technologies can be divided into two categories. The first encompasses sequencing-based methods, which use next-generation sequencing to identify barcodes encoded with spatial information. The second type is imaging-based methods, which use microscopy to directly visualize mRNA *in situ*. Alternatively, spatial methods can also be divided into targeted and untargeted methods, depending on whether probes are used.

Despite the appeal of spatial approaches, their adoption is often limited by the high upfront costs and ongoing reagent expenses. Benchmarking studies have been instrumental in helping scientists evaluate the advantages and disadvantages of different technologies, providing guidance on selecting the most suitable solutions for their specific needs,^43^^,^^44^^,^^45^^,^^46^ but these will need to be continuously updated to reflect the latest advancements and options available.

### Sequencing-based methods

The first methodological approach that allowed spatial profiling of the transcriptome was analyzing user-defined regions like cubes (voxels) of tissue, individual tissue sections, areas selected through laser capture microdissection or optical marking^47^ using microarrays, RNA-seq, or scRNA-seq. Examples of technologies that used such an approach are voxelation,^48^ Tomo-seq,^49^ Geo-seq,^50^ STRP-seq,^51^ NICHE-seq,^52^ SPACECAT,^53^ and ZipSeq,^54^ and commercially available solutions include NanoString Technologies’ GeoMx Digital Spatial Profiler.^55^ In 2016, the advent of Spatial Transcriptomics^56^—developed by a company with the same namesake and subsequently acquired by 10× Genomics—marked a significant milestone in the field of sequencing-based spatial omics methods. Eventually becoming known as the Visium platform, this technique utilized a spatially barcoded array, which consisted of a microarray with probes of known sequences positioned at known locations, to capture the mRNA from a permeabilized tissue section using its oligo(dT) probes. Thus, the spatial resolution of capture is dependent on the capture array, area of the capture spots, and the distance between these capture spots. Since then, the quest for higher resolution and improved performance has spurred the development of several new protocols.

Slide-seq,^57^ commercialized by Curio Bioscience under the name Curio Seeker, introduced a novel method of capturing mRNA on a surface covered with spatially barcoded beads, offering enhanced resolution. Despite its advantages, the bead-based technology can sometimes lead to reduced mRNA capture efficiency. This technology was subsequently adapted into Slide-DNA-seq,^58^ enabling the capture of spatially resolved DNA sequences from intact tissue sections; however, this technology has not yet been commercially offered.

Another notable advancement from BGI Genomics is Stereo-seq,^59^ which employs DNA nanoballs for transcript capture at ultra-high resolution, allowing for higher-resolution spatial mapping. Finally, AtlasXomics has developed Deterministic Barcode in Tissue sequencing (DBiT-seq), which offers unbiased profiling of the transcriptome and epigenome at the cellular level,^60^^,^^61^ thus initiating a spatial epigenomics market.

Open-source solutions are also becoming an emerging part of the spatial technology landscape. For example, Open-ST^62^ provides a cost-efficient and scalable spatial transcriptomics platform by retrofitting Illumina NovaSeq S4 flow cells—allowing for the capture of transcripts at subcellular resolution—and thus facilitates the 3D reconstruction of tissue using sequential tissue sections. Given the high cost of spatial transcriptomics, such cost-effective innovations offer viable, alternative, high-cost spatial transcriptomic platforms.

### Imaging-based methods

**Figure 2:**
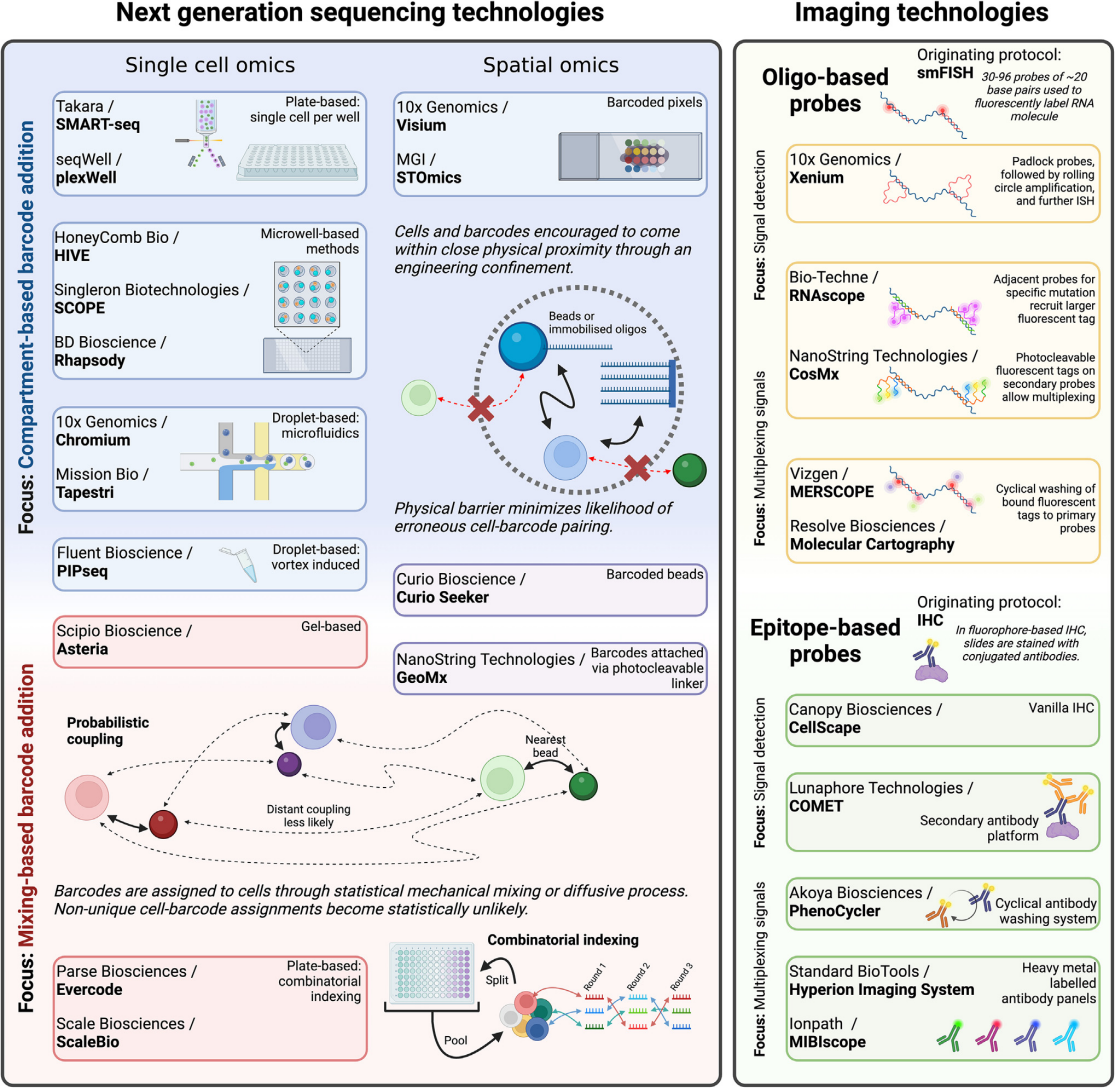
Single-cell and spatial omic platform characterization using experimental motifs common to similar technologies

From the first report of detection of RNA molecules in tissues by *in situ* hybridization using radioactive tritium in 1969, the field has undergone significant advancements.^63^ Non-radioactive fluorescent dyes and a highly multiplexed approach were developed to measure transcripts with high detection efficiency and resolution of subcellular spatial localization called single-molecule fluorescent *in situ* hybridization^64^ (smFISH). From this originating method, a variety of improvements were posed largely to increase flexibility and signal-to-noise ratio and to allow for higher multiplexing capacity (i.e., detecting a greater number of diverse transcripts). While the body of this review focuses on the detection of transcripts, many of these methods have corresponding epitope-based methods for quantitative proteomics, illustrated in Figure 2.

To enhance transcript detection with higher signal-to-noise ratios, two principal strategies have emerged. The first strategy mirrors the principles of immunohistochemistry, where the use of secondary fluorescent probes amplifies an initial signal. The approach is exemplified by the Bio-Techne RNAscope and NanoString’s CosMX platform, which employs multiple probes to target a single RNA molecule, thereby increasing signal strength, followed by a non-enzymatic probe amplification, increasing the signal-to-noise ratio of transcript detection events. NanoString’s introduction of CosMx Spatial Molecular Imager represents another significant development in this field, allowing for high multiplexing capacity via the use of UV-cleaved fluorescent dyes. This feature allows for sequential labeling and imaging and reversible labeling of RNA targets, thereby permitting sequential imaging and analysis of various targets within the same tissue sample.

*In situ* sequencing (ISS) methods represent a second strategy in which the spatial locations of transcripts can be probed via the use of barcoded padlock probes before enzymatic ligation and rolling circle amplification (RCA) chemistry.^65^ A notable example is the 10× Genomics Xenium platform.^66^ This method enzymatically amplifies the circularized padlock probes before the generated rolling circle products can be fluorescently labeled through successive rounds of hybridization. Both strategies—multi-probe and multi-step hybridization—offer distinct advantages in increasing the detectability of gene expression in spatial transcriptomics with high signal-to-noise ratio using lower-magnification objectives.

Building upon smFISH,^67^ where transcripts are detected with high sensitivity but lack multiplexing capacity, highly multiplexed methods such as multiplexed error robust fluorescence *in situ* hybridization (MERFISH) have been designed to address and overcome multiplexing issues associated with smFISH while simultaneously preserving high detection sensitivity.^68^ MERFISH, which was commercialized by Vizgen as the MERSCOPE platform, utilizes combinatorial labeling and error robust barcoding; the latter is especially beneficial, as it significantly reduces the risk of transcript misidentification, a prevalent issue in high-throughput assays. Another technology working in a similar manner is Resolve Bioscience’s Molecular Cartography platform, which enables up to 100 transcripts to be detected with subcellular resolution.

## Trends

As we researched the aforementioned companies, several broad conceptual themes have emerged. In the case of technologies reliant on next-generation sequencing, there is often a distinction in the process whereby cells are assigned a barcode: either cells and barcode are matched through optimizing some engineering process to bring cells and barcodes into close proximity or methods rely on the underlying disorder of the system to achieve a unique cell-barcode assignment between cells and barcodes. While this is not a strict distinction, it is instructive in that solutions that “compartmentalize” cells and barcodes typically rest on greater levels of sophisticated engineering. Imaging technologies can be subdivided based on the underlying probe system: using either oligonucleotides or epitopes. In Figure 2, we show how different commercial solutions fit into this schema. We also highlight the compromise that imaging platforms face: one must prioritize between signal detection and the ability to multiplex (i.e., detecting more diverse transcripts); see Tables S1 and S2 for more details.

### Computational pipelines

After sequencing a library generated from one of the next-generation-sequencing-based single-cell or spatial omic technologies, one needs a mechanism to make inferences with regards to the experimental data in question. Historically, this process has been divided into preprocessing and downstream analysis. For each technology, a preferred preprocessing method has often, but not always, been recommended by the platform originators; however, downstream analysis is typically custom, with a large open-source effort to create and distribute new methods (usually written in R or Python) for consumption by the broader scientific community. A few platform-specific analysis and visualization tools with simple user interfaces that do not require any coding knowledge have also been developed.

Preprocessing of scRNA-seq data describes the computational process of estimating the number of distinct molecules (potentially including a splicing status) arising from each gene within each quantified cell from the read output generated by the sequencer (FASTQ file). The resulting count matrix forms the basis for all downstream analyses, and, thus, any biases and errors in the preprocessing pipeline will affect the acquired results. In addition, the considerable size of the raw sequencing data and the size of the genome make the preprocessing computationally intensive. This double challenge, achieving high accuracy while keeping required computational efforts manageable, has sparked the development of a variety of tools and pipelines. Yet, overall, preprocessing can be broken down into a sequence of steps. It starts with raw data quality control, which allows the diagnosis of any problems with library preparation or sequencing. The most complex step is then the alignment of the reads to a reference genome. This is then followed by cell-barcode identification and correction and an estimation of molecule counts through deduplication of unique molecular identifiers.

Running these pipelines and adapting them to the specific requirements of each single-cell technology is a considerable effort that requires expert knowledge, motivating industry-backed bioinformatic solutions, detailed in Table S3. These can consist of either downloadable software packages or integrated cloud solutions tailored for datasets that, depending on sequencing depth and the number of cells sequenced, are too large to be handled on personal computers.

Because of the market dominance of 10× Genomics, the most notable of such solutions are the Cell Ranger and Space Ranger packages. It is a one-stop solution for all datasets obtained using 10× Genomics library preparation protocols. Under the hood, Cell Ranger uses the Spliced Transcripts Alignment to a Reference (STAR) aligner,^69^ a high-accuracy but relatively memory-intensive alignment software. STAR is open-source software and is used widely in both bulk and scRNA-seq analysis as a versatile, powerful, and time-tested mapping solution. Over the last few years, single-cell functionality has been added to (i.e., built into) STAR in the form of the STARsolo suite.^70^ Besides STARsolo, other options without industry sponsorship are available, including two main pseudoalignment- or quasi-mapping-based platforms: kallisto/bustools^71^ and salmon/alevin-fry.^72^ Pseudoalignment can achieve significant increases in computational efficiency by avoiding a base-level alignment. Instead kallisto assigns read-to-transcript mapping probabilities based on hash lookups on a transcript k-mer Bruijn graph.^73^ These platforms offer the benefit of flexibility, being able to accommodate a wide range of library structures (i.e., any cell barcode and unique molecular identifier [UMI] length and position in the reads), and can resolve differential transcript usage. A comprehensive review^74^ covers additional details about different aligners, as well as the intricacies of the other pipeline steps.

With regards to downstream analysis, various computational analysis pipelines exist to analyze count matrices generated from the preprocessing pipelines and produce standard outputs of interest, e.g., filtering of low-quality cells, dead cells, empty droplets, cell doublets, cell type annotations, differential expression testing, etc. Two packages have emerged as class leaders in their respective programming languages: Seurat^75^ (R) and SCANPY^76^ (Python). The “recipes” one uses to obtain such outputs are relatively customizable, and best-practice pipelines^77^ have been established. In contrast, pipelines to analyze processed spatial genomics data are less standardized but are being built as extensions to existing packages, e.g., Squidpy,^78^ which is part of the scverse collection of packages. We direct the interested reader to one of the many reviews of said pipelines,^77^ which details the scope of possible inferences (outside of the scope for this commentary).

For imaging-based spatial transcriptomics platforms, there are additional challenges including image stitching, registration, illumination correction, background subtraction, single-cell segmentation, and basic cell phenotyping. Vendor-provided platforms include Horizon (Lunaphore), MACS iQ View (Miltenyi), and Multiplex Analysis Viewer (Akoya Biosciences). For more complex analyses, researchers can explore open-source tools^79^^,^^80^ or specialized third-party software providers. These providers, like Enable Medicine, Visiopharm, Indica Labs, Aspect Analytics, and ariadne.ai, offer more sophisticated functionalities, typically tailored for highly multiplexed antibody-based imaging.

Finally, given the inherently heterogeneous nature of spatial data, handling such multiplexed data effectively will require methods that better support data integration. Adopting open and universal data frameworks, such as those proposed in SpatialData,^81^ will be necessary to effectively manage the wave of incoming public data generated from spatial omics. This will allow for seamless integration of sequencing data (both spatial and non-spatial) with imaging-based spatial transcriptomics methods, which offer significant complementary and additive power in uncovering new biological insights. For example, by combining single-cell and spatial technologies, researchers have been able to profile gene expression in large formalin-fixed paraffin-embedded human breast cancer samples,^66^ enabling exploration of molecular differences between distinct tumor regions.

### Single-cell epigenomics methods

Cellular identities are determined through the interplay between various epigenetic layers that collectively regulate transcriptional program. These layers include (1) DNA methylation, (2) chromatin accessibility, (3) histone modification and transcription factor binding, and (4) 3D genome organization. Current commercial solutions enable the study of (1) and (2) at single-cell resolution: 10× Genomics and Bio-Rad have scATAC-seq offerings, and Scale Biosciences has recently launched a single-cell DNA methylation kit. However, no current commercial kit enables the measurement of histone mark and transcription factor (TF) binding or 3D genome organization at single-cell resolution. While DNA methylation and chromatin accessibility provide important information about genome regulatory states, these features do not fully inform on cellular fates. First of all, accessible chromatin regions (identified with ATAC-seq) can present different chromatin states characterized by specific histone marks that can lead to completely different regulatory outcomes.^82^^,^^83^ For instance both active and bivalent chromatin states are accessible but may have opposite effects on gene expression.^84^ In addition, motif analysis of accessible chromatin regions are not enough to identify the TFs driving cellular identities for two main reasons: (1) ATAC-seq will detect TF footprints of TFs that are not expressed, and (2) TFs frequently access the genome via non-canonical motifs,^85^ which are missing in the TF motif databases used in ATAC-seq analysis. Moreover, chromatin factors (CFs), shown as key drivers of cellular states and diseases,^86^^,^^87^ bind DNA in a motif-agnostic manner, and their functions cannot be deconvoluted from ATAC-seq studies. Therefore, direct profiling of TFs and CFs is key to identifying the drivers of cellular states and diseases. Finally, specific 3D genome topologies sustain cell-type-specific genome regulatory circuits, and detailed 3D genome maps have enabled non-coding disease-associated DNA variants with their target genes.^88^

A precise dissection of epigenetic states with single-cell resolution is of particular interest for diseases with altered differentiation patterns caused by corrupted epigenetic regulation, with examples including many cancers (especially blood malignancies) and neurodevelopmental disease.^89^^,^^90^ In recent years, several academic labs have developed technologies that enable profiling histone mark and TF binding at single-cell resolution.^16^^,^^91^^,^^92^^,^^93^ Many of these methods require highly specialized equipment and training; however, some of these methods can be run by adapting commercial solutions and, therefore, easily implemented in standard laboratories. For instance, the Henikoff and Castelo-Branco labs have leveraged the 10× Genomics scATAC-seq kits to develop single-cell CUT&Tag methods that allow histone mark profiling at single-cell resolution.^94^ These approaches were further improved in late 2022, with the development of single-cell multi-modal chromatin profiling methods (nanoCT and NTT-seq^19^) that enable the simultaneous profiling of three histone marks per single cell. Importantly, these single-cell multi-modal chromatin maps enable one to study the connections between complex chromatin states and corrupted cellular states across diverse diseases, potentially enabling the development of novel epigenetic therapies. Of note, NTT-seq has been released as a starter kit by the Center for Integrated Cellular Analysis (https://www.multimodalintegration.org/tech). Finally, single-cell 3D genome profiling methods^95^^,^^96^ (scMicro-C and scNanoHi-C) still require specialized training and equipment, but we expect that key companies in the field, Cantata Bio and Arima Genomics, will soon develop commercial versions of these protocols.

### Total RNA-seq

Most high-throughput (10×, combinatorial indexing) and low-throughput (SMART-seq,^97^^,^^98^^,^^99^ Cel-seq2^100^) single-cell methods aim to resolve gene expression values across single-cell populations. However, such methods rely mostly on poly(A) capture to uniquely extract mRNA molecules from the pool of RNA molecules in the cell (mostly non-informative ribosomal RNAs). However, this reliance on poly(A) enrichment precludes the detection of some non-coding RNAs (some long non-coding RNAs, piRNA, miRNA, small nuclear RNA, small nucleolar RNA, etc.), which crucially report on gene regulation as well as transcription dynamics. Moreover, since most single-cell methods use short-read next-generation sequencing to determine gene expression, they typically tag only the 3′ or 5′ end of the mRNA molecule. This approach inherently limits the analytical scope, focusing on depth at the expense of breadth.^101^ While it enables analysis of specific gene expression levels, it precludes comprehensive evaluation of alternative promoter usage and splicing analyses. These latter aspects are vital for understanding cell-type-specific gene function.^102^^,^^103^ The depth of information gained from 3′ or 5′ end tagging is valuable, yet the breadth lost in overlooking alternative splicing and promoter usage is a significant trade-off in these single-cell methodologies.^104^

To remediate this, a host of new emerging methods have focused on recovering larger parts of the transcriptome from single cells. First, low-throughput SMART-seq methods^97^^,^^98^^,^^99^ rely on full transcript capture using tagmentation, which adds splicing information to gene expression values. However, the inability to barcode all tagmented fragments precludes high-throughput implementation, and the methods do not enable the detection of non-polyadenylated non-coding RNA species. Other emerging methods, termed total RNA-seq methods, depict a more accurate picture of a cell’s transcriptome and generally rely on total RNA tagging achieved either using via enzymatic tailing (VASA-seq,^14^ SMART-seq-total,^105^ and MATQ-seq^106^) or random hexamer hybridization (RAMDA-seq^107^). Because ribosomal RNAs are considered non-informative, they are later depleted using sequence-specific depletion—via duplex degradation using complementary probes or using Cas9.^108^

Total RNA-seq methods have, for example, been used to determine the alternative splicing landscape during mammalian organogenesis at single-cell resolution (VASA-seq^14^) or to observe transcriptome fluctuations in subcellular compartments (single synapses using MATQ-Drop^15^). Further, total RNA-seq methods have been deployed to the single-cell Visium framework by adding a polyadenylation step to tag non-polyadenylated species, shining light on the localized non-coding RNA distribution during skeletal muscle regeneration (STRS^109^). However, it is important to note that these high-throughput total RNA-seq methods predominantly rely on in-house protocols. This reliance can act as a barrier to widespread adoption in the broader scientific community, as compared to more established methods. Additionally, the customization of these protocols often leads to variability in data quality and reproducibility across different laboratories. Moreover, the complexity and resource-intensive nature of these in-house techniques may limit their use to well-funded or specialized research groups, potentially hindering the democratization of this approach in diverse research settings.

### Intracellular protein detection

The integration of cell-surface-protein measurements with scRNA-seq through methods such as CITE-seq^110^ have greatly enhanced the understanding of cellular protein markers and functional proteins on the cell surface, providing a more comprehensive view of cell state. However, initial protein measurements based on live-cell input were limited to cell-surface proteins and unable to measure intracellular protein levels in tandem with the transcriptome, which provides an additional critical layer of cell-state information that underlies cell function. Detecting diverse classes of intracellular proteins, such as TFs, protein post-translational modifications (PTMs) that compose cell-signaling networks, cell-cycle regulators, and other functional proteins, is crucial to interpret cell state and function. Following the CITE-seq (cell-surface Protein and RNA) and ECCITE-seq^111^ (cell-surface protein, RNA and CRISPR guides) approaches, a host of technologies have been developed to probe intracellular protein abundance using intracellular-protein-targeting antibodies tagged with barcoded oligonucleotides (–antibody-derived tags [ADTs]). Two key protocol innovations in the development of these technologies have been the optimization of appropriate fixation and permeabilization with respective downstream scRNA-seq/scATAC-seq technology protocols and the development of effective blocking steps to reduce nonspecific signal using single-stranded nucleic acids, negatively charged polymers, or bacterial single-stranded DNA-binding proteins to block intracellular features of the cell that might nonspecifically bind to the ADTs. Droplet-based technologies such as ASAP-seq,^112^ based on 10× ATAC-seq, were extended to intracellular proteins based on formaldehyde fixation and permeabilization with NP-40. Similarly, inCITE-seq^113^ extended 10× scRNA-seq to detect TFs leveraging FA-NT (formaldehyde, NP-40, Tween 20, and glacial acetic acid) simultaneous fixation and permeabilization. In parallel, plate-based approaches, such as RAID,^114^ further extended to a droplet-based system with QuRIE-seq^115^ were also developed to identify phospho-protein PTMs to measure intracellular cell signaling using a reversible fixation-based DSP/SPDP and Triton X-100.

Recent advances using droplet-based approaches have focused on simultaneously detecting intracellular protein, ATAC-seq, and RNA based on the 10× Multiome protocol. NEAT-seq^116^ deployed an ADT panel targeting TFs and developed an enhanced blocking process using the *E. coli* single-stranded DNA-binding protein EcoSSB to bind single-stranded DNA ADTs, prevent nonspecific binding, and enhance signal to noise. Phospho-seq^117^ extended ASAP-seq using EcoSSB blocking, deploying an ADT panel targeting TFs and protein PTM phosphorylation sites in combination with scATAC-seq, integrating RNA measurements computationally via bridge integration. 10× Genomics has recently adapted several features of these protocols into the probe-based 10× Genomics Flex assay, based on fixed cell input that offers protocols to integrate intracellular protein measurements with ADTs.

Other foundational scRNA-seq technologies, such as combinatorial indexing approaches like SPLiT-seq,^36^ depend on fixed and permeabilized cells and are well suited to be integrated with ADT-based intracellular protein detection. SIGNAL-seq^118^ has extended SPLiT-seq to measure intracellular proteins and protein PTMs to map intracellular signaling with transcriptome, leveraging the intrinsic strengths of combinatorial indexing, such as increased scale across both cell number and conditions per assay in an instrument-independent and cost-effective manner.

### Bacterial whole-genome or transcriptome sequencing

Eukaryotic cells are by far the most highly sequenced organisms due to their direct translational implications toward improving human health. However, prokaryotes and, more specifically, bacteria are emerging as new targets for single-cell sequencing campaigns, mainly whole-genome or transcriptome sequencing (as well as epigenetic). Primarily, genome sequencing efforts are designed for surveillance purposes, where a strain can be matched to genetic background such as antibiotic resistance cassettes, whereas transcriptome endeavors aim to elucidate phenotypic responses to drug perturbations. Recent advances have demonstrated that highly multiplexed spatial transcriptomics in bacteria is possible,^119^ highlighting the rapid progress in this area. Three main avenues for high-throughput sequencing have been derived: (1) droplet-based assays (SIC-seq,^120^ Microbe-seq^121^), (2) combinatorial indexing methods (PETRI-seq,^122^ microSPLiT-seq^123^), or (3) a combination of droplet and combinatorial indexing (BacDrop^124^). Similar to total RNA-seq methods, barcoding strategies have been devised to circumvent reliance on poly(A) barcoding methodologies, mainly via tagmentation and barcoding in droplets, barcoded random hexamer capture, or RNA tailing. Though still in its early stages, bacterial single-cell sequencing is a rapidly emerging field. It promises to yield novel genotypic and phenotypic insights, significantly enhancing our understanding of host-pathogen interactions. This advancement not only holds potential in medical research but also has broad implications for agricultural science.

### Long-read sequencing

The human genome consists of approximately 20,000 protein-coding genes, which speculatively encode more than 100,000 distinct proteins.^125^ With the inclusion of T cell receptor, B cell receptor, and antibody diversity, the tally of unique proteins potentially extends into the millions. The diversity of the human proteome exceeds the genome, in part because of alternative splicing and recombination events, which can create many more combinations of substrates from the same gene or combinations of gene segments, respectively.

Though full-length, single-cell sequencing methods like SMART-seq2/3^98^ enable transcript-level analysis, their reliance on short-read data demands convoluted computational analyses to derive meaningful information.^126^ This complexity is partly due to the intricate gene models associated with certain genes. Currently, droplet-based single-cell sequencing techniques predominantly capture either the 3′ or 5′ end of a transcript and employ short-read sequencing methods. This means that we are only capturing a fraction of the cellular information in which to infer the phenotype of a cell. This limitation poses a significant challenge in detecting gene rearrangements and alternative splicing, leaving a significant chunk of cellular information untapped and consequently impeding the accurate inference of cellular phenotype.

Long-read sequencing platforms, such as PacBio and Oxford Nanopore Technologies (ONT), provide end-to-end sequencing of mRNA. This enables a more exhaustive exploration of complete isoforms, RNA splicing events, single-nucleotide polymorphisms, structural variation, imprinting, and measurement of chimeric transcripts at the single-cell level.^127^ Despite these capabilities, the widespread adoption of long-read technologies faces several technical obstacles. Key challenges include low-throughput and sequencing inaccuracies that affect cell assignment and PCR artifact removal. Moreover, the high cost and complexity of these technologies, along with substantial data analysis requirements, further impede their broad utilization. Long-read sequencing adoption is hindered by the need for staff upskilling and the implementation of methods to manage the voluminous and computationally intensive data. Compared to short-read methods, these requirements make long-read sequencing less accessible for many laboratories. Despite these challenges, long-read sequencing is invaluable for unraveling complex genomic regions, identifying novel isoforms, and studying genetic diseases with intricate mutation patterns. In cases where detailed genomic information is crucial, the depth and accuracy of long-read sequencing make it an indispensable tool, outweighing its limitations.

Historically, PacBio’s sequencing throughput has lagged behind the ONT platform.^128^ For single-cell applications, this limited throughput allows for only a small number of cells per PacBio flow cell, thus obstructing comprehensive transcriptomic profiling at the single-cell level. The advent of the MAS-Iso-seq protocol,^129^ a technique that concatenates cDNAs into single molecules, has made it possible to sequence thousands of single cells using PacBio sequencing, reaching a level comparable to that achievable with ONT sequencing. This advancement allows for an increased number of cells to be sequenced per study and an enhanced read depth per cell, facilitating superior isoform expression profiling.

When contrasting ONT and PacBio sequencing accuracy, PacBio has traditionally been recognized for its higher accuracy. Nonetheless, both have higher sequencing error rates than the Illumina platform.^130^ However, if a user requires qualitative single-cell data, sequencing using either long-read platform alone is sufficient. However, because of the higher error rates, applying long-read sequencing to single cells remains a challenge for accurate quantification. This is not just related to the sequencing errors but also to PCR errors, which are higher for long-read platforms because of the higher library input requirements.^131^ Initial strategies to tackle these issues for long-read sequencing involved merging the data from libraries sequenced simultaneously using Illumina sequencing.^128^^,^^132^^,^^133^ The higher-accuracy Illumina sequencing data serve as a guide to correct the barcode and UMI regions of the long-read sequencing data. Unfortunately, this leads to only 40%–60% of reads being effectively recovered, and the necessity for dual sequencing platforms rendered this approach less desirable.^133^ Alternative methods, such as computational strategies^134^ have been explored for error correction. However, computational solutions alone do not sufficiently address the high PCR errors^131^ associated with long-read technologies. Nevertheless, errors can be effectively corrected using UMIs built with homoblocks of nucleotides.^101^ This provides an error-correcting solution that enables absolute counting of sequenced molecules, eliminating the need for dual sequencing. However, no commercial single-cell platform currently supports effective error correction for long-read technologies, making dual sequencing the commonly used approach for accurate quantification.

Despite the increased adoption of long-read technologies for single-cell applications, broader utilization of these technologies in single-cell biology faces significant challenges. Notably, issues with accuracy and usability persist. Additionally, a substantial computational hurdle exists due to the larger file sizes associated with long-read sequencing data and the scarcity of methods tailored to their unique characteristics. Therefore, for wider adoption of this technology, future efforts must focus on developing specialized laboratory methods, protocols, and computational tools specifically designed to handle, process, and maximize the potential of long-read sequencing data.

### Metabolomics sequencing

Metabolomics, as the youngest of the omics sciences, plays a crucial role by offering a snapshot of the cell’s biochemical activities, thereby serving as a readout closest to the phenotype. The metabolome, reflecting the dynamic interactions between the cell and its environment, varies significantly even among genetically identical cells.^135^ Factors such as diet and disease states, like cancer, can alter a cell’s metabolome,^136^ influencing processes such as immune evasion, angiogenesis, and metastasis in the tumor microenvironment.

Single-cell metabolomics has become a powerful tool in this landscape, particularly for identifying subpopulations within complex tissues such as tumors. However, single-cell metabolomics faces several challenges. The rapid dynamism of the metabolome can complicate sample collection, as metabolite levels can change swiftly,^137^ potentially altering the metabolome’s native state. The complexity of metabolites, which vastly outnumbers that of genomic or transcriptome elements, poses challenges in their accurate identification and quantification. The METASPACE platform exemplifies the advances in metabolite identification through its algorithmic approach, facilitating the interpretation of imaging mass spectrometry (MS) data.^138^ This tool enables researchers to process complex spectral data efficiently, accelerating the generation of meaningful molecular insights.

Finally, spatial metabolomics adds another dimension by mapping metabolite distributions across tissues, providing a means to visualize and quantify metabolic activity *in situ*. This approach integrates metabolomic data with the spatial context of the cell and its microenvironment, enabling the elucidation of metabolic networks across health and disease.^139^

### Protein sequencing

A leap in protein sequencing would usher in new frontiers in understanding complex biology, much like how RNA-seq revolutionized our understanding of gene expression. While DNA sequencing has made it possible to map generic variations, these variations often do not correlate with phenotypic observable traits. Given that only a portion of mRNA is translated into protein, and considering the significant variations in RNA half-lives, protein sequencing could provide a more accurate insight into a cell’s current phenotypic state. Conventional MS-based proteomics, which has been the cornerstone for protein measurement, falls short in providing a high resolution of protein analysis. Analysis of proteins is also complicated because the 20,000 genes encode many transcripts, which can be spliced into a diversity of proteoforms that may include millions of variants as a result of further PTMs. Characterizing these proteoforms is crucial for a deeper understanding of biological processes and disease mechanisms, especially in contexts where gene expression does not fully explain phenotypic outcomes. For example, in cancer biology, understanding the proteomic changes can provide insights into tumor progression and treatment responses that genomic data alone may not reveal. Similarly, in neurodegenerative diseases, the study of specific protein aggregates is essential for understanding disease pathology.

MS remains a staple of protein quantification, and several methods have been developed toward supporting single-cell applications. Recent advancements in the miniaturization of sample processing workflows, combined with a multiplexed strategy, have made single-cell proteomics possible by improving sample preparation efficiency and allowing unique tagging of proteins from individual cells, resulting in the ability to quantify more than 1,000 proteins from a single-cell. However, other technologies such as DNA-facilitated protein sequencing, massive parallel Edman degradation, protein fingerprinting using FRET, and biological and solid-state nanopores have shown great promise as potential solutions for supporting single-cell applications.^140^

Protein sequencing, while still in its infancy, has made strides thanks to innovative technology solutions and a handful of commercial platforms that focus on single-molecule protein sequencing. Quantum-SI is one such provider, employing their time domain sequencing method.^141^ This approach involves immobilizing peptides onto semiconductor chips, which are then recognized and interacted with by fluorescently labeled N-terminal amino acid (NAA) recognizers and aminopeptidases. Once a recognizer binds, fluorescence is measured and the amino acid is cleaved, preparing the system for the next round of binding and measurement. A similar technique involving fluoro-sequencing is provided by Erisyon.^140^ Their process commences with proteosome digestion, then several of the amino acid types on these peptides are then selectively and covalently labeled with fluorophores that uniquely identify each peptide. The peptides are then immobilized on a glass slide and fluorescence is measured. Nautilus Biotechnology has also pioneered a novel fluoro-sequencing approach, which utilizes fluorescent affinity probes that bind specific protein motifs. A cyclic process of applying and then removing these probes generates a unique “fingerprint” in which machine-learning analysis can convert into protein identities and quantification. Additionally, Pixelgen Technologies has commercialized a method called Molecular Pixelation (MPX),^142^ an optics-free, DNA-sequence-based approach for spatial proteomics. MPX utilizes antibody-oligonucleotide conjugates (AOCs) and DNA-based molecular pixels to map protein distributions at the single-cell level. This innovative technique allows for the study of protein spatial arrangements without the limitations of traditional fluorescence microscopy, enabling more detailed insights into cellular processes.

Several publications have demonstrated the feasibility of conducting single-cell proteomics using MS approaches.^143^ Beyond the challenges in throughput and sensitivity, there is also the issue of interpretability and the depth of biological information that can be derived from these studies. The complexity of data obtained from single-cell MS-based proteomics is substantial, often presenting challenges in data processing and analysis. This complexity can hinder the ability to draw clear and actionable biological insights. Moreover, the depth of biological information is frequently limited by the inability of current MS techniques to comprehensively detect and quantify the full range of proteins, particularly low-abundance and transient proteins, within a single cell. Therefore, while single-cell MS-based proteomics is a promising field, there is a need for advancements in technology and analytical methods to improve not only the throughput and sensitivity but also the interpretability and biological information content of the data obtained.

Single-molecule sequencing technology, with its lower input demands, offers the potential for delivering higher-throughput and enhanced peptide resolution at the single-cell level. Nevertheless, the practical adaptation of these techniques to single-cell approaches in their current form presents a considerable challenge. In parallel, approaches are being developed, such as the use of DNA-tagged proteins sequenced through nanopores.^144^^,^^145^ These emerging methods represent a promising direction in the field, potentially overcoming some of the limitations of current single-cell proteomic techniques.

## Closing remarks

Two macro trends are likely to define genomic technology development in the next decade: a steady increase in US healthcare costs per capita and the drastic decrease in the cost of sequencing.^146^ Across every therapeutic area, we find that broadly defined disease-associated phenotypes are likely multiple actionable endotypes that should be treated through a precision medicine lens. This evolving future is being shaped by companies that are currently commercializing single-cell and spatial omic technologies, either in a research setting or as a diagnostic. The integration of these technologies, typically using machine learning,^147^ is crucial in revolutionizing the drug discovery and development process, enabling a more targeted and efficient approach. Furthermore, initiatives like scTrends are pivotal in enabling us to understand how the drug development landscape is adapting to these changes. They offer valuable perspectives on which technologies and business models are likely to succeed in the coming years. However, the transformational potential of genomic technologies in shaping healthcare and drug development is not yet fully conveyed or understood due to the constant state of flux. There is therefore a need for a clearer articulation of how these trends wilel impact the future of healthcare, the viability of various technologies, and effective business strategies in this rapidly evolving field.

## Consortia

The scTrends Consortium includes all authors in addition to Eli M. Carrami, Rebecca McIntyre, Casey Benjamin Swerner, Edith M. Hessel, Chantriolnt-Andreas Kapourani, Cristian Regep, and Charles E.S. Roberts.

## Acknowledgments

A.P.C. is supported by a Medical Research Council Career Development Fellowship (MR/V010182/1).

## Declaration of interests

J.D.J., J.W.O., and A.P.C. are inventors on patent applications filed by the University of Cambridge (via Cambridge Enterprise), the University College London Business, and Oxford University Innovations, respectively. J.W.O., A.V.Z., T.G., J.C., L.R.-M., C.E.S.R., A.K.S., and J.P.T.-K. receive compensation from Relation Therapeutics. P.C. and A.P.C. are employees of Caereleus Genomics. B.S.N. and N.D.K. receive compensation from OMAPiX. M.V. and J.L. are scientific consultants for 10× Genomics. J.M. receives compensation from Skyhawk Therapeutics and previously worked as a scientific consultant for Scipio Bioscience. K.S. receives compensation from Roche. D.M. receives compensation from scDiscoveries. H.L., S.M.S., and C.M.L. receive compensation from spatialist. A.K.S. reports compensation for consulting and/or scientific advisory board membership from Honeycomb Biotechnologies, Cellarity, Ochre Bio, Relation Therapeutics, Bio-Rad Laboratories, IntrECate Biotherapeutics, Passkey Therapeutics, Fog Pharma, and Dahlia Biosciences, which are unrelated to this work. D.S. reports funding from Cellzome, a GSK company, and received honorariums from Immunai, Noetik, Alpenglow, and Lunaphore.

## Supplemental information

Table S1. Detailed review of commercial single-cell technology specifications, related to Figure 2

Table S2. Detailed review of commercial spatial omic technology specifications, related to Figure 2

Table S3. Summary of industry-maintained software/pipelines for commercial single-cell and spatial omics technology, related to Figure 2

## References

[1] Marx V. (2021). Method of the Year: spatially resolved transcriptomics. Nat. Methods.

[2] (2020). Method of the Year 2019: Single-cell multimodal omics. Nat. Methods.

[3] Papalexi E., Satija R. (2018). Single-cell RNA sequencing to explore immune cell heterogeneity. Nat. Rev. Immunol..

[4] Dixit A., Parnas O., Li B., Chen J., Fulco C.P., Jerby-Arnon L., Marjanovic N.D., Dionne D., Burks T., Raychowdhury R. (2016). Perturb-Seq: Dissecting Molecular Circuits with Scalable Single-Cell RNA Profiling of Pooled Genetic Screens. Cell.

[5] Datlinger P., Rendeiro A.F., Schmidl C., Krausgruber T., Traxler P., Klughammer J., Schuster L.C., Kuchler A., Alpar D., Bock C. (2017). Pooled CRISPR screening with single-cell transcriptome readout. Nat. Methods.

[6] Jaitin D.A., Weiner A., Yofe I., Lara-Astiaso D., Keren-Shaul H., David E., Salame T.M., Tanay A., van Oudenaarden A., Amit I. (2016). Dissecting Immune Circuits by Linking CRISPR-Pooled Screens with Single-Cell RNA-Seq. Cell.

[7] De Jonghe J., Opzoomer J.W., Vilas-Zornoza A., Crane P., Nilges B.S., Vicari M., Lee H., Lara-Astiaso D., Gross T., Morf J. (2024). A community effort to track commercial single-cell and spatial ’omic technologies and business trends. Nat. Biotechnol..

[8] Dai X., Shen L. (2022). Advances and Trends in Omics Technology Development. Front. Med..

[9] Peidli S., Green T.D., Shen C., Gross T., Min J., Garda S., Yuan B., Schumacher L.J., Taylor-King J.P., Marks D.S. (2024). scPerturb: harmonized single-cell perturbation data. Nat. Methods.

[10] Zilionis R., Nainys J., Veres A., Savova V., Zemmour D., Klein A.M., Mazutis L. (2017). Single-cell barcoding and sequencing using droplet microfluidics. Nat. Protoc..

[11] Macosko E.Z., Basu A., Satija R., Nemesh J., Shekhar K., Goldman M., Tirosh I., Bialas A.R., Kamitaki N., Martersteck E.M. (2015). Highly Parallel Genome-wide Expression Profiling of Individual Cells Using Nanoliter Droplets. Cell.

[12] De Rop F.V., Ismail J.N., Bravo González-Blas C., Hulselmans G.J., Flerin C.C., Janssens J., Theunis K., Christiaens V.M., Wouters J., Marcassa G. (2022). Hydrop enables droplet-based single-cell ATAC-seq and single-cell RNA-seq using dissolvable hydrogel beads. eLife.

[13] De Jonghe J., Kaminski T.S., Morse D.B., Tabaka M., Ellermann A.L., Kohler T.N., Amadei G., Handford C.E., Findlay G.M., Zernicka-Goetz M. (2023). spinDrop: a droplet microfluidic platform to maximise single-cell sequencing information content. Nat. Commun..

[14] Salmen F., De Jonghe J., Kaminski T.S., Alemany A., Parada G.E., Verity-Legg J., Yanagida A., Kohler T.N., Battich N., van den Brekel F. (2022). High-throughput total RNA sequencing in single cells using VASA-seq. Nat. Biotechnol..

[15] Niu M., Cao W., Wang Y., Zhu Q., Luo J., Wang B., Zheng H., Weitz D.A., Zong C. (2023). Droplet-based transcriptome profiling of individual synapses. Nat. Biotechnol..

[16] Rotem A., Ram O., Shoresh N., Sperling R.A., Goren A., Weitz D.A., Bernstein B.E. (2015). Single-cell ChIP-seq reveals cell subpopulations defined by chromatin state. Nat. Biotechnol..

[17] Grosselin K., Durand A., Marsolier J., Poitou A., Marangoni E., Nemati F., Dahmani A., Lameiras S., Reyal F., Frenoy O. (2019). High-throughput single-cell ChIP-seq identifies heterogeneity of chromatin states in breast cancer. Nat. Genet..

[18] Bartosovic M., Castelo-Branco G. (2023). Multimodal chromatin profiling using nanobody-based single-cell CUT&Tag. Nat. Biotechnol..

[19] Stuart T., Hao S., Zhang B., Mekerishvili L., Landau D.A., Maniatis S., Satija R., Raimondi I. (2023). Nanobody-tethered transposition enables multifactorial chromatin profiling at single-cell resolution. Nat. Biotechnol..

[20] Xu Z., Zhang T., Chen H., Zhu Y., Lv Y., Zhang S., Chen J., Chen H., Yang L., Jiang W. (2023). High-throughput single nucleus total RNA sequencing of formalin-fixed paraffin-embedded tissues by snRandom-seq. Nat. Commun..

[21] Regev A., Teichmann S.A., Lander E.S., Amit I., Benoist C., Birney E., Bodenmiller B., Campbell P., Carninci P., Clatworthy M. (2017). The Human Cell Atlas. eLife.

[22] Lareau C.A., Duarte F.M., Chew J.G., Kartha V.K., Burkett Z.D., Kohlway A.S., Pokholok D., Aryee M.J., Steemers F.J., Lebofsky R., Buenrostro J.D. (2019). Droplet-based combinatorial indexing for massive-scale single-cell chromatin accessibility. Nat. Biotechnol..

[23] Gierahn T.M., Wadsworth M.H., Hughes T.K., Bryson B.D., Butler A., Satija R., Fortune S., Love J.C., Shalek A.K. (2017). Seq-Well: portable, low-cost RNA sequencing of single cells at high throughput. Nat. Methods.

[24] Huang S., Ziegler C.G.K., Austin J., Mannoun N., Vukovic M., Ordovas-Montanes J., Shalek A.K., Von Andrian U.H. (2021). Lymph nodes are innervated by a unique population of sensory neurons with immunomodulatory potential. Cell.

[25] Shalek A.K., Satija R., Adiconis X., Gertner R.S., Gaublomme J.T., Raychowdhury R., Schwartz S., Yosef N., Malboeuf C., Lu D. (2013). Single-cell transcriptomics reveals bimodality in expression and splicing in immune cells. Nature.

[26] Prakadan S.M., Shalek A.K., Weitz D.A. (2017). Scaling by shrinking: empowering single-cell “omics” with microfluidic devices. Nat. Rev. Genet..

[27] Han X., Wang R., Zhou Y., Fei L., Sun H., Lai S., Saadatpour A., Zhou Z., Chen H., Ye F. (2018). Mapping the Mouse Cell Atlas by Microwell-Seq. Cell.

[28] Bose S., Wan Z., Carr A., Rizvi A.H., Vieira G., Pe’er D., Sims P.A. (2015). Scalable microfluidics for single-cell RNA printing and sequencing. Genome Biol..

[29] Gogoi P., Sepehri S., Zhou Y., Gorin M.A., Paolillo C., Capoluongo E., Gleason K., Payne A., Boniface B., Cristofanilli M. (2016). Development of an Automated and Sensitive Microfluidic Device for Capturing and Characterizing Circulating Tumor Cells (CTCs) from Clinical Blood Samples. PLoS One.

[30] Dura B., Choi J.-Y., Zhang K., Damsky W., Thakral D., Bosenberg M., Craft J., Fan R. (2019). scFTD-seq: freeze-thaw lysis based, portable approach toward highly distributed single-cell 3′ mRNA profiling. Nucleic Acids Res..

[31] DeKosky B.J., Ippolito G.C., Deschner R.P., Lavinder J.J., Wine Y., Rawlings B.M., Varadarajan N., Giesecke C., Dörner T., Andrews S.F. (2013). High-throughput sequencing of the paired human immunoglobulin heavy and light chain repertoire. Nat. Biotechnol..

[32] Hughes T.K., Wadsworth M.H., Gierahn T.M., Do T., Weiss D., Andrade P.R., Ma F., De Andrade Silva B.J., Shao S., Tsoi L.C. (2020). Second-Strand Synthesis-Based Massively Parallel scRNA-Seq Reveals Cellular States and Molecular Features of Human Inflammatory Skin Pathologies. Immunity.

[33] Cao J., Packer J.S., Ramani V., Cusanovich D.A., Huynh C., Daza R., Qiu X., Lee C., Furlan S.N., Steemers F.J. (2017). Comprehensive single-cell transcriptional profiling of a multicellular organism. Science.

[34] Cusanovich D.A., Daza R., Adey A., Pliner H.A., Christiansen L., Gunderson K.L., Steemers F.J., Trapnell C., Shendure J. (2015). Multiplex single-cell profiling of chromatin accessibility by combinatorial cellular indexing. Science.

[35] Vitak S.A., Torkenczy K.A., Rosenkrantz J.L., Fields A.J., Christiansen L., Wong M.H., Carbone L., Steemers F.J., Adey A. (2017). Sequencing thousands of single-cell genomes with combinatorial indexing. Nat. Methods.

[36] Rosenberg A.B., Roco C.M., Muscat R.A., Kuchina A., Sample P., Yao Z., Graybuck L.T., Peeler D.J., Mukherjee S., Chen W. (2018). Single-cell profiling of the developing mouse brain and spinal cord with split-pool barcoding. Science.

[37] Srivatsan S.R., McFaline-Figueroa J.L., Ramani V., Saunders L., Cao J., Packer J., Pliner H.A., Jackson D.L., Daza R.M., Christiansen L. (2020). Massively multiplex chemical transcriptomics at single-cell resolution. Science.

[38] Xu Z., Sziraki A., Lee J., Zhou W., Cao J. (2024). Dissecting key regulators of transcriptome kinetics through scalable single-cell RNA profiling of pooled CRISPR screens. Nat. Biotechnol..

[39] Baldwin M., Buckley C.D., Guilak F., Hulley P., Cribbs A.P., Snelling S. (2023). A roadmap for delivering a human musculoskeletal cell atlas. Nat. Rev. Rheumatol..

[40] Van Den Brink S.C., Sage F., Vértesy Á., Spanjaard B., Peterson-Maduro J., Baron C.S., Robin C., Van Oudenaarden A. (2017). Single-cell sequencing reveals dissociation-induced gene expression in tissue subpopulations. Nat. Methods.

[41] Gomariz A., Helbling P.M., Isringhausen S., Suessbier U., Becker A., Boss A., Nagasawa T., Paul G., Goksel O., Székely G. (2018). Quantitative spatial analysis of haematopoiesis-regulating stromal cells in the bone marrow microenvironment by 3D microscopy. Nat. Commun..

[42] Williams C.G., Lee H.J., Asatsuma T., Vento-Tormo R., Haque A. (2022). An introduction to spatial transcriptomics for biomedical research. Genome Med..

[43] Rademacher A., Huseynov A., Bortolomeazzi M., Wille S.J., Schumacher S., Sant P., Keitel D., Okonechnikov K., Ghasemi D.R., Pajtler K.W. (2024). Comparison of spatial transcriptomics technologies using tumor cryosections. bioRxiv.

[44] Cook D.P., Jensen K.B., Wise K., Roach M.J., Dezem F.S., Ryan N.K., Zamojski M., Vlachos I.S., Knott S.R.V., Butler L.M. (2023). A Comparative Analysis of Imaging-Based Spatial Transcriptomics Platforms. bioRxiv.

[45] Hartman A., Satija R. (2024). Comparative analysis of multiplexed in situ gene expression profiling technologies. eLife.

[46] You Y., Fu Y., Li L., Zhang Z., Jia S., Lu S., Ren W., Liu Y., Xu Y., Liu X. (2024). Systematic comparison of sequencing-based spatial transcriptomic methods. Nat. Methods.

[47] Moses L., Pachter L. (2022). Museum of spatial transcriptomics. Nat. Methods.

[48] Brown V.M., Ossadtchi A., Khan A.H., Yee S., Lacan G., Melega W.P., Cherry S.R., Leahy R.M., Smith D.J. (2002). Multiplex three-dimensional brain gene expression mapping in a mouse model of Parkinson’s disease. Genome Res..

[49] Junker J.P., Noël E.S., Guryev V., Peterson K.A., Shah G., Huisken J., McMahon A.P., Berezikov E., Bakkers J., van Oudenaarden A. (2014). Genome-wide RNA Tomography in the Zebrafish Embryo. Cell.

[50] Peng G., Suo S., Chen J., Chen W., Liu C., Yu F., Wang R., Chen S., Sun N., Cui G. (2016). Spatial Transcriptome for the Molecular Annotation of Lineage Fates and Cell Identity in Mid-gastrula Mouse Embryo. Dev. Cell.

[51] Schede H.H., Schneider C.G., Stergiadou J., Borm L.E., Ranjak A., Yamawaki T.M., David F.P.A., Lönnerberg P., Tosches M.A., Codeluppi S., La Manno G. (2021). Spatial tissue profiling by imaging-free molecular tomography. Nat. Biotechnol..

[52] Medaglia C., Giladi A., Stoler-Barak L., De Giovanni M., Salame T.M., Biram A., David E., Li H., Iannacone M., Shulman Z., Amit I. (2017). Spatial reconstruction of immune niches by combining photoactivatable reporters and scRNA-seq. Science.

[53] Genshaft A.S., Ziegler C.G.K., Tzouanas C.N., Mead B.E., Jaeger A.M., Navia A.W., King R.P., Mana M.D., Huang S., Mitsialis V. (2021). Live cell tagging tracking and isolation for spatial transcriptomics using photoactivatable cell dyes. Nat. Commun..

[54] Hu K.H., Eichorst J.P., McGinnis C.S., Patterson D.M., Chow E.D., Kersten K., Jameson S.C., Gartner Z.J., Rao A.A., Krummel M.F. (2020). ZipSeq: barcoding for real-time mapping of single cell transcriptomes. Nat. Methods.

[55] Merritt C.R., Ong G.T., Church S.E., Barker K., Danaher P., Geiss G., Hoang M., Jung J., Liang Y., McKay-Fleisch J. (2020). Multiplex digital spatial profiling of proteins and RNA in fixed tissue. Nat. Biotechnol..

[56] Ståhl P.L., Salmén F., Vickovic S., Lundmark A., Navarro J.F., Magnusson J., Giacomello S., Asp M., Westholm J.O., Huss M. (2016). Visualization and analysis of gene expression in tissue sections by spatial transcriptomics. Science.

[57] Rodriques S.G., Stickels R.R., Goeva A., Martin C.A., Murray E., Vanderburg C.R., Welch J., Chen L.M., Chen F., Macosko E.Z. (2019). Slide-seq: A scalable technology for measuring genome-wide expression at high spatial resolution. Science..

[58] Zhao T., Chiang Z.D., Morriss J.W., LaFave L.M., Murray E.M., Del Priore I., Meli K., Lareau C.A., Nadaf N.M., Li J. (2022). Spatial genomics enables multi-modal study of clonal heterogeneity in tissues. Nature.

[59] Chen A., Liao S., Cheng M., Ma K., Wu L., Lai Y., Qiu X., Yang J., Xu J., Hao S. (2022). Spatiotemporal transcriptomic atlas of mouse organogenesis using DNA nanoball-patterned arrays. Cell.

[60] Deng Y., Bartosovic M., Kukanja P., Zhang D., Liu Y., Su G., Enninful A., Bai Z., Castelo-Branco G., Fan R. (2022). Spatial-CUT&Tag: Spatially resolved chromatin modification profiling at the cellular level. Science.

[61] Zhang D., Deng Y., Kukanja P., Agirre E., Bartosovic M., Dong M., Ma C., Ma S., Su G., Bao S. (2023). Spatial epigenome–transcriptome co-profiling of mammalian tissues. Nature.

[62] Schott M., León-Periñán D., Splendiani E., Strenger L., Licha J.R., Pentimalli T.M., Schallenberg S., Alles J., Samut Tagliaferro S., Boltengagen A. (2024). Open-ST: High-resolution spatial transcriptomics in 3D. Cell.

[63] Pardue M.L., Gall J.G. (1969). MOLECULAR HYBRIDIZATION OF RADIOACTIVE DNA TO THE DNA OF CYTOLOGICAL PREPARATIONS. Proc. Natl. Acad. Sci. USA.

[64] Femino A.M., Fay F.S., Fogarty K., Singer R.H. (1998). Visualization of single RNA transcripts in situ. Science.

[65] Ke R., Mignardi M., Pacureanu A., Svedlund J., Botling J., Wählby C., Nilsson M. (2013). In situ sequencing for RNA analysis in preserved tissue and cells. Nat. Methods.

[66] Janesick A., Shelansky R., Gottscho A.D., Wagner F., Williams S.R., Rouault M., Beliakoff G., Morrison C.A., Oliveira M.F., Sicherman J.T. (2023). High resolution mapping of the tumor microenvironment using integrated single-cell, spatial and in situ analysis. Nat. Commun..

[67] Safieddine A., Coleno E., Lionneton F., Traboulsi A.-M., Salloum S., Lecellier C.-H., Gostan T., Georget V., Hassen-Khodja C., Imbert A. (2023). HT-smFISH: a cost-effective and flexible workflow for high-throughput single-molecule RNA imaging. Nat. Protoc..

[68] Chen K.H., Boettiger A.N., Moffitt J.R., Wang S., Zhuang X. (2015). Spatially resolved, highly multiplexed RNA profiling in single cells. Science.

[69] Dobin A., Davis C.A., Schlesinger F., Drenkow J., Zaleski C., Jha S., Batut P., Chaisson M., Gingeras T.R. (2013). STAR: ultrafast universal RNA-seq aligner. Bioinformatics.

[70] Kaminow B., Yunusov D., Dobin A. (2021). STARsolo: accurate, fast and versatile mapping/quantification of single-cell and single-nucleus RNA-seq data. bioRxiv.

[71] Melsted P., Booeshaghi A.S., Liu L., Gao F., Lu L., Min K.H.J., Da Veiga Beltrame E., Hjörleifsson K.E., Gehring J., Pachter L. (2021). Modular, efficient and constant-memory single-cell RNA-seq preprocessing. Nat. Biotechnol..

[72] He D., Zakeri M., Sarkar H., Soneson C., Srivastava A., Patro R. (2022). Alevin-fry unlocks rapid, accurate and memory-frugal quantification of single-cell RNA-seq data. Nat. Methods.

[73] Compeau P.E.C., Pevzner P.A., Tesler G. (2011). How to apply de Bruijn graphs to genome assembly. Nat. Biotechnol..

[74] Heumos L., Schaar A.C., Lance C., Litinetskaya A., Drost F., Zappia L., Lücken M.D., Strobl D.C., Henao J., Curion F. (2023). Best practices for single-cell analysis across modalities. Nat. Rev. Genet..

[75] Satija R., Farrell J.A., Gennert D., Schier A.F., Regev A. (2015). Spatial reconstruction of single-cell gene expression data. Nat. Biotechnol..

[76] Wolf F.A., Angerer P., Theis F.J. (2018). SCANPY: large-scale single-cell gene expression data analysis. Genome Biol..

[77] Luecken M.D., Theis F.J. (2019). Current best practices in single-cell RNA-seq analysis: a tutorial. Mol. Syst. Biol..

[78] Palla G., Spitzer H., Klein M., Fischer D., Schaar A.C., Kuemmerle L.B., Rybakov S., Ibarra I.L., Holmberg O., Virshup I. (2022). Squidpy: a scalable framework for spatial omics analysis. Nat. Methods.

[79] Bankhead P., Loughrey M.B., Fernández J.A., Dombrowski Y., McArt D.G., Dunne P.D., McQuaid S., Gray R.T., Murray L.J., Coleman H.G. (2017). QuPath: Open source software for digital pathology image analysis. Sci. Rep..

[80] Schapiro D., Sokolov A., Yapp C., Chen Y.-A., Muhlich J.L., Hess J., Creason A.L., Nirmal A.J., Baker G.J., Nariya M.K. (2022). MCMICRO: a scalable, modular image-processing pipeline for multiplexed tissue imaging. Nat. Methods.

[81] Marconato L., Palla G., Yamauchi K.A., Virshup I., Heidari E., Treis T., Vierdag W.-M., Toth M., Stockhaus S., Shrestha R.B. (2024). SpatialData: an open and universal data framework for spatial omics. Nat. Methods.

[82] Shema E., Jones D., Shoresh N., Donohue L., Ram O., Bernstein B.E. (2016). Single-molecule decoding of combinatorially modified nucleosomes. Science.

[83] Kimura H. (2013). Histone modifications for human epigenome analysis. J. Hum. Genet..

[84] Macrae T.A., Fothergill-Robinson J., Ramalho-Santos M. (2023). Regulation, functions and transmission of bivalent chromatin during mammalian development. Nat. Rev. Mol. Cell Biol..

[85] Samee M.A.H. (2023). Noncanonical binding of transcription factors: time to revisit *specificity*. Mol. Biol. Cell.

[86] Valencia A.M., Sankar A., Van Der Sluijs P.J., Satterstrom F.K., Fu J., Talkowski M.E., Vergano S.A.S., Santen G.W.E., Kadoch C. (2023). Landscape of mSWI/SNF chromatin remodeling complex perturbations in neurodevelopmental disorders. Nat. Genet..

[87] Lara-Astiaso D., Goñi-Salaverri A., Mendieta-Esteban J., Narayan N., Del Valle C., Gross T., Giotopoulos G., Beinortas T., Navarro-Alonso M., Aguado-Alvaro L.P. (2023). In vivo screening characterizes chromatin factor functions during normal and malignant hematopoiesis. Nat. Genet..

[88] Orozco G., Schoenfelder S., Walker N., Eyre S., Fraser P. (2022). 3D genome organization links non-coding disease-associated variants to genes. Front. Cell Dev. Biol..

[89] Agrawal-Singh S., Bagri J., Sakakini N., Huntly B.J.P. (2023). A guide to epigenetics in leukaemia stem cells. Mol. Oncol..

[90] Berson A., Nativio R., Berger S.L., Bonini N.M. (2018). Epigenetic Regulation in Neurodegenerative Diseases. Trends Neurosci..

[91] Xie Y., Zhu C., Wang Z., Tastemel M., Chang L., Li Y.E., Ren B. (2023). Droplet-based single-cell joint profiling of histone modifications and transcriptomes. Nat. Struct. Mol. Biol..

[92] Yeung J., Florescu M., Zeller P., De Barbanson B.A., Wellenstein M.D., Van Oudenaarden A. (2023). scChIX-seq infers dynamic relationships between histone modifications in single cells. Nat. Biotechnol..

[93] Rang F.J., De Luca K.L., De Vries S.S., Valdes-Quezada C., Boele E., Nguyen P.D., Guerreiro I., Sato Y., Kimura H., Bakkers J., Kind J. (2022). Single-cell profiling of transcriptome and histone modifications with EpiDamID. Mol. Cell.

[94] Bartosovic M., Kabbe M., Castelo-Branco G. (2021). Single-cell CUT&Tag profiles histone modifications and transcription factors in complex tissues. Nat. Biotechnol..

[95] Wu, H., Zhang, J., Tan, L., and Xie, X.S. Extruding transcription elongation loops observed in high-resolution single-cell 3D genomes. Preprint at bioRxiv 10.1101/2023.02.18.529096.

[96] Li W., Lu J., Lu P., Gao Y., Bai Y., Chen K., Su X., Li M., Liu J., Chen Y. (2023). scNanoHi-C: a single-cell long-read concatemer sequencing method to reveal high-order chromatin structures within individual cells. Nat. Methods.

[97] Hagemann-Jensen M., Ziegenhain C., Chen P., Ramsköld D., Hendriks G.-J., Larsson A.J.M., Faridani O.R., Sandberg R. (2020). Single-cell RNA counting at allele and isoform resolution using Smart-seq3. Nat. Biotechnol..

[98] Picelli S., Faridani O.R., Björklund A.K., Winberg G., Sagasser S., Sandberg R. (2014). Full-length RNA-seq from single cells using Smart-seq2. Nat. Protoc..

[99] Ramsköld D., Luo S., Wang Y.-C., Li R., Deng Q., Faridani O.R., Daniels G.A., Khrebtukova I., Loring J.F., Laurent L.C. (2012). Full-length mRNA-Seq from single-cell levels of RNA and individual circulating tumor cells. Nat. Biotechnol..

[100] Hashimshony T., Senderovich N., Avital G., Klochendler A., de Leeuw Y., Anavy L., Gennert D., Li S., Livak K.J., Rozenblatt-Rosen O. (2016). CEL-Seq2: sensitive highly-multiplexed single-cell RNA-Seq. Genome Biol..

[101] Philpott M., Watson J., Thakurta A., Brown T., Brown T., Oppermann U., Cribbs A.P. (2021). Nanopore sequencing of single-cell transcriptomes with scCOLOR-seq. Nat. Biotechnol..

[102] Joglekar A., Hu W., Zhang B., Narykov O., Diekhans M., Marrocco J., Balacco J., Ndhlovu L.C., Milner T.A., Fedrigo O. (2024). Single-cell long-read sequencing-based mapping reveals specialized splicing patterns in developing and adult mouse and human brain. Nat. Neurosci..

[103] Kumari P., Kaur M., Dindhoria K., Ashford B., Amarasinghe S.L., Thind A.S. (2024). Advances in long-read single-cell transcriptomics. Hum. Genet..

[104] Zajac N., Zhang Q., Bratus-Neuschwander A., Qi W., Bolck H.A., Karakulak T., Oltra T.C., Moch H., Kahraman A., Rehrauer H. (2024). Comparison of Single-cell Long-read and Short-read Transcriptome Sequencing of Patient-derived Organoid Cells of ccRCC: Quality Evaluation of the MAS-ISO-seq Approach. bioRxiv.

[105] Isakova A., Neff N., Quake S.R. (2021). Single-cell quantification of a broad RNA spectrum reveals unique noncoding patterns associated with cell types and states. Proc. Natl. Acad. Sci. USA.

[106] Sheng K., Cao W., Niu Y., Deng Q., Zong C. (2017). Effective detection of variation in single-cell transcriptomes using MATQ-seq. Nat. Methods.

[107] Hayashi T., Ozaki H., Sasagawa Y., Umeda M., Danno H., Nikaido I. (2018). Single-cell full-length total RNA sequencing uncovers dynamics of recursive splicing and enhancer RNAs. Nat. Commun..

[108] Loi D.S.C., Yu L., Wu A.R. (2021). Effective ribosomal RNA depletion for single-cell total RNA-seq by scDASH. PeerJ.

[109] McKellar D.W., Mantri M., Hinchman M.M., Parker J.S.L., Sethupathy P., Cosgrove B.D., De Vlaminck I. (2023). Spatial mapping of the total transcriptome by in situ polyadenylation. Nat. Biotechnol..

[110] Stoeckius M., Hafemeister C., Stephenson W., Houck-Loomis B., Chattopadhyay P.K., Swerdlow H., Satija R., Smibert P. (2017). Simultaneous epitope and transcriptome measurement in single cells. Nat. Methods.

[111] Mimitou E.P., Cheng A., Montalbano A., Hao S., Stoeckius M., Legut M., Roush T., Herrera A., Papalexi E., Ouyang Z. (2019). Multiplexed detection of proteins, transcriptomes, clonotypes and CRISPR perturbations in single cells. Nat. Methods.

[112] Mimitou E.P., Lareau C.A., Chen K.Y., Zorzetto-Fernandes A.L., Hao Y., Takeshima Y., Luo W., Huang T.-S., Yeung B.Z., Papalexi E. (2021). Scalable, multimodal profiling of chromatin accessibility, gene expression and protein levels in single cells. Nat. Biotechnol..

[113] Chung H., Parkhurst C.N., Magee E.M., Phillips D., Habibi E., Chen F., Yeung B.Z., Waldman J., Artis D., Regev A. (2021). Joint single-cell measurements of nuclear proteins and RNA in vivo. Nat. Methods.

[114] Gerlach J.P., Van Buggenum J.A.G., Tanis S.E.J., Hogeweg M., Heuts B.M.H., Muraro M.J., Elze L., Rivello F., Rakszewska A., Van Oudenaarden A. (2019). Combined quantification of intracellular (phospho-)proteins and transcriptomics from fixed single cells. Sci. Rep..

[115] Rivello F., Van Buijtenen E., Matuła K., Van Buggenum J.A.G.L., Vink P., Van Eenennaam H., Mulder K.W., Huck W.T.S. (2021). Single-cell intracellular epitope and transcript detection reveals signal transduction dynamics. Cell Rep. Methods.

[116] Chen A.F., Parks B., Kathiria A.S., Ober-Reynolds B., Goronzy J.J., Greenleaf W.J. (2022). NEAT-seq: simultaneous profiling of intra-nuclear proteins, chromatin accessibility and gene expression in single cells. Nat. Methods.

[117] Blair J.D., Hartman A., Zenk F., Dalgarno C., Treutlein B., Satija R. (2023). Phospho-seq: Integrated, multi-modal profiling of intracellular protein dynamics in single cells. bioRxiv.

[118] Opzoomer J.W., O’Sullivan R., Sufi J., Madsen R., Qin X., Basiarz E., Tape C.J. (2024). SIGNAL-seq: Multimodal Single-cell Inter- and Intra-cellular Signalling Analysis. bioRxiv.

[119] Sarfatis A., Wang Y., Twumasi-Ankrah N., Moffitt J.R. (2024). Highly Multiplexed Spatial Transcriptomics in Bacteria. bioRxiv.

[120] Lan F., Demaree B., Ahmed N., Abate A.R. (2017). Single-cell genome sequencing at ultra-high-throughput with microfluidic droplet barcoding. Nat. Biotechnol..

[121] Zheng W., Zhao S., Yin Y., Zhang H., Needham D.M., Evans E.D., Dai C.L., Lu P.J., Alm E.J., Weitz D.A. (2022). High-throughput, single-microbe genomics with strain resolution, applied to a human gut microbiome. Science.

[122] Blattman S.B., Jiang W., Oikonomou P., Tavazoie S. (2020). Prokaryotic single-cell RNA sequencing by in situ combinatorial indexing. Nat. Microbiol..

[123] Kuchina A., Brettner L.M., Paleologu L., Roco C.M., Rosenberg A.B., Carignano A., Kibler R., Hirano M., DePaolo R.W., Seelig G. (2021). Microbial single-cell RNA sequencing by split-pool barcoding. Science.

[124] Ma P., Amemiya H.M., He L.L., Gandhi S.J., Nicol R., Bhattacharyya R.P., Smillie C.S., Hung D.T. (2023). Bacterial droplet-based single-cell RNA-seq reveals antibiotic-associated heterogeneous cellular states. Cell.

[125] Uhlén M., Fagerberg L., Hallström B.M., Lindskog C., Oksvold P., Mardinoglu A., Sivertsson Å., Kampf C., Sjöstedt E., Asplund A. (2015). Tissue-based map of the human proteome. Science.

[126] Booeshaghi A.S., Yao Z., Van Velthoven C., Smith K., Tasic B., Zeng H., Pachter L. (2021). Isoform cell-type specificity in the mouse primary motor cortex. Nature.

[127] Gupta P., O’Neill H., Wolvetang E.J., Chatterjee A., Gupta I. (2024). Advances in single-cell long-read sequencing technologies. NAR Genom. Bioinform..

[128] Gupta I., Collier P.G., Haase B., Mahfouz A., Joglekar A., Floyd T., Koopmans F., Barres B., Smit A.B., Sloan S.A. (2018). Single-cell isoform RNA sequencing characterizes isoforms in thousands of cerebellar cells. Nat. Biotechnol..

[129] Al’Khafaji A.M., Smith J.T., Garimella K.V., Babadi M., Popic V., Sade-Feldman M., Gatzen M., Sarkizova S., Schwartz M.A., Blaum E.M. (2024). High-throughput RNA isoform sequencing using programmed cDNA concatenation. Nat. Biotechnol..

[130] Amarasinghe S.L., Su S., Dong X., Zappia L., Ritchie M.E., Gouil Q. (2020). Opportunities and challenges in long-read sequencing data analysis. Genome Biol..

[131] Sun J., Philpott M., Loi D., Li S., Monteagudo-Mesas P., Hoffman G., Robson J., Mehta N., Gamble V., Brown T. (2024). Correcting PCR amplification errors in unique molecular identifiers to generate accurate numbers of sequencing molecules. Nat. Methods.

[132] Wang Q., Boenigk S., Boehm V., Gehring N.H., Altmueller J., Dieterich C. (2021). Single-cell transcriptome sequencing on the Nanopore platform with ScNapBar. RNA.

[133] Lebrigand K., Magnone V., Barbry P., Waldmann R. (2020). High throughput error corrected Nanopore single cell transcriptome sequencing. Nat. Commun..

[134] Smith T., Heger A., Sudbery I. (2017). UMI-tools: modeling sequencing errors in Unique Molecular Identifiers to improve quantification accuracy. Genome Res..

[135] Qiu S., Cai Y., Yao H., Lin C., Xie Y., Tang S., Zhang A. (2023). Small molecule metabolites: discovery of biomarkers and therapeutic targets. Signal Transduct. Target. Ther..

[136] Martínez-Reyes I., Chandel N.S. (2021). Cancer metabolism: looking forward. Nat. Rev. Cancer.

[137] Ali A., Davidson S., Fraenkel E., Gilmore I., Hankemeier T., Kirwan J.A., Lane A.N., Lanekoff I., Larion M., McCall L.-I. (2022). Single cell metabolism: current and future trends. Metabolomics.

[138] Alexandrov T., Ovchinnikova K., Palmer A., Kovalev V., Tarasov A., Stuart L., Nigmetzianov R., Fay D., Gaudin M., Key METASPACE contributors (2019). METASPACE: A community-populated knowledge base of spatial metabolomes in health and disease. bioRxiv.

[139] Hu T., Allam M., Cai S., Henderson W., Yueh B., Garipcan A., Ievlev A.V., Afkarian M., Beyaz S., Coskun A.F. (2023). Single-cell spatial metabolomics with cell-type specific protein profiling for tissue systems biology. Nat. Commun..

[140] Alfaro J.A., Bohländer P., Dai M., Filius M., Howard C.J., Van Kooten X.F., Ohayon S., Pomorski A., Schmid S., Aksimentiev A. (2021). The emerging landscape of single-molecule protein sequencing technologies. Nat. Methods.

[141] Reed B.D., Meyer M.J., Abramzon V., Ad O., Adcock P., Ahmad F.R., Alppay G., Ball J.A., Beach J., Belhachemi D. (2022). Real-time dynamic single-molecule protein sequencing on an integrated semiconductor device. Science.

[142] Karlsson F., Kallas T., Thiagarajan D., Karlsson M., Schweitzer M., Navarro J.F., Leijonancker L., Geny S., Pettersson E., Rhomberg-Kauert J. (2024). Molecular pixelation: spatial proteomics of single cells by sequencing. Nat. Methods.

[143] Bennett H.M., Stephenson W., Rose C.M., Darmanis S. (2023). Single-cell proteomics enabled by next-generation sequencing or mass spectrometry. Nat. Methods.

[144] Yu L., Kang X., Li F., Mehrafrooz B., Makhamreh A., Fallahi A., Foster J.C., Aksimentiev A., Chen M., Wanunu M. (2023). Unidirectional single-file transport of full-length proteins through a nanopore. Nat. Biotechnol..

[145] Motone K., Nivala J. (2023). Not if but when nanopore protein sequencing meets single-cell proteomics. Nat. Methods.

[146] Simmons S.K., Lithwick-Yanai G., Adiconis X., Oberstrass F., Iremadze N., Geiger-Schuller K., Thakore P.I., Frangieh C.J., Barad O., Almogy G. (2023). Mostly natural sequencing-by-synthesis for scRNA-seq using Ultima sequencing. Nat. Biotechnol..

[147] Taylor-King J.P., Bronstein M., Roblin D. (2024). The Future of Machine Learning Within Target Identification: Causality, Reversibility, and Druggability. Clin. Pharmacol. Ther..

